# Interleukin-17–producing T cells are enriched in the joints of children with arthritis, but have a reciprocal relationship to regulatory T cell numbers

**DOI:** 10.1002/art.23291

**Published:** 2008-03

**Authors:** Kiran Nistala, Halima Moncrieffe, Katy R Newton, Hemlata Varsani, Patricia Hunter, Lucy R Wedderburn

**Affiliations:** University College LondonLondon, UK

## Abstract

**Objective:**

To identify interleukin-17 (IL-17)–producing T cells from patients with juvenile idiopathic arthritis (JIA), and investigate their cytokine production, migratory capacity, and relationship to Treg cells at sites of inflammation, as well as to test the hypothesis that IL-17+ T cell numbers correlate with clinical phenotype in childhood arthritis.

**Methods:**

Flow cytometry was used to analyze the phenotype, cytokine production, and chemokine receptor expression of IL-17–producing T cells in peripheral blood and synovial fluid mononuclear cells from 36 children with JIA, in parallel with analysis of forkhead box P3 (FoxP3)–positive Treg cells. Migration of IL-17+ T cells toward CCL20 was assessed by a Transwell assay. Synovial tissue was analyzed by immunohistochemistry for IL-17 and IL-22.

**Results:**

IL-17+ T cells were enriched in the joints of children with JIA as compared with the blood of JIA patients (*P* = 0.0001) and controls (*P* = 0.018) and were demonstrated in synovial tissue. IL-17+ T cell numbers were higher in patients with extended oligoarthritis, the more severe subtype of JIA, as compared with patients with persistent oligoarthritis, the milder subtype (*P* = 0.046). Within the joint, there was an inverse relationship between IL-17+ T cells and FoxP3+ Treg cells (r = 0.61, *P* = 0.016). IL-17+,CD4+ T cells were uniformly CCR6+ and migrated toward CCL20, but synovial IL-17+ T cells had variable CCR4 expression. A proportion of IL-17+ synovial T cells produced IL-22 and interferon-γ.

**Conclusion:**

This study is the first to define the frequency and characteristics of “Th17” cells in JIA. We suggest that these highly proinflammatory cells contribute to joint pathology, as indicated by relationships with clinical phenotypes, and that the balance between IL-17+ T cells and Treg cells may be critical to outcome.

Human autoimmune diseases, including inflammatory arthritis in both adults and children, have been previously associated with a Th1 polarized response at the site of inflammation (1–3). A new subset of effector T cells that produce interleukin-17 (IL-17) has recently been characterized (4–6). In mice, these Th17 cells appear to be driven by the transcription factor retinoic acid–related orphan receptor γt (RORγt) and to be developmentally and functionally distinct from interferon-γ (IFNγ)–producing type 1 (Th1) and IL-4– producing type 2 (Th2) helper T cells (7–9). Aberrant Th17 cell responses have been linked with autoimmune disease in several animal models, including collagen-induced arthritis and experimental autoimmune encephalomyelitis, and it has been increasingly proposed that Th17 cells, rather than Th1 cells, may be central to disease pathogenesis (10,11).

The differentiation of murine Th17 cells from naive T cells appears to require transforming growth factor β (TGFβ) and IL-6 (8). Thus, the conditions for generating these potent effector cells are related to those required to generate regulatory T cells, namely, TGFβ alone, whereas IL-6 and possibly other inflammatory cytokines can provide the key switch from generation of Treg cells to Th17 cells (12). The requirements for the generation of human Th17 cells in the physiologic context may be more dependent on IL-6 and IL-1 (13,14), but what regulates the balance between Th17 cells and Treg cells in health or disease has not yet been fully elucidated.

The IL-17 family of cytokines, which includes the T cell–derived proinflammatory cytokines IL-17A and IL-17F, has been implicated in the pathogenesis of rheumatoid arthritis (RA) and, more recently, juvenile idiopathic arthritis (JIA). IL-17 is produced spontaneously by explanted synovial tissue from RA patients, has been found at high levels in synovial fluid (15,16), and has many downstream effects that mimic the pathology of inflammatory arthritis. In JIA, IL-17 is increased in patients with active disease as compared with those in remission (17). IL-17 promotes a proinflammatory cytokine milieu in the joint, stimulating the production of IL-1 and tumor necrosis factor α (TNFα) from macrophages (18), and synergizes with these to increase IL-6 and IL-8 production from RA synoviocytes (19,20), the latter being important in neutrophil recruitment. IL-17 also promotes bone erosion through the up-regulation of RANKL (15), a key regulator of osteoclast neogenesis. RANKL has been demonstrated in high levels in JIA (21,22) and RA (23,24). Downstream effects of IL-17 include production of metalloproteinases and proteoglycan breakdown, leading to cartilage destruction (25,26).

Initial reports demonstrated Th17 cells in healthy adults that express homing receptors and antigen specificities distinct from those of Th1 and Th2 cells (27). In that study, a proportion of IL-17–producing T cells also expressed IFNγ. T cells making both IL-17 and IFNγ have also been observed in the synovial cells of RA patients (28) and patients with Lyme arthritis (29). However, the role and character of Th17 cells in human arthritis and their relationship to Treg cells have not been fully investigated to date.

JIA is a heterogeneous group of childhood arthropathies (30) that offers a clinical paradigm by which to examine the Treg cell–Th17 cell interplay at the site of inflammation. We and other investigators have shown that regulatory T cells are enriched in the synovial fluid of JIA patients (31,32) and that these Treg cells are present at significantly higher numbers in patients with a milder clinical phenotype (persistent oligoarticular JIA) than in those with a more severe form of arthritis (extended oligoarticular JIA) (31). We have also shown a marked enrichment of IFNγ-producing T cells that express high levels of the chemokine receptors CCR5 and CXCR3 in the JIA joint (3) and that chemokines (CCL5, CCL3, and CXCL10) that attract Th1 cells are enriched in JIA synovial fluid (33). We have now investigated whether Th17 cells can be demonstrated at the site of inflammation in JIA and, if so, how they relate to the Treg cell and Th1 cell populations, as well as to the clinical disease phenotype.

The aims of this study were to identify and characterize human IL-17–producing T cells from patients with JIA, the most common rheumatologic inflammatory autoimmune disease of childhood. We tested the hypothesis that the numbers of IL-17+ T cells correlate with the clinical phenotype in childhood arthritis. We explored the relationship between these Th17 cells and FoxP3+ Treg cells at sites of inflammation. We then investigated the production of other cytokines by IL-17+ T cells in JIA, in particular, IFNγ, IL-4, and IL-22, their expression of chemokine receptors, and their capacity to migrate to the CCR6 ligand CCL20. We believe this to be the first study of IL-17+ T cells and their relationship to FoxP3+ Treg cells in human arthritis.

## PATIENTS AND METHODS

### Patients and samples

Samples of peripheral blood and synovial fluid from 36 children (28 females and 8 males) who met the International League of Associations for Rheumatology criteria for JIA (30) and 11 healthy controls (7 females and 4 males) were included in the study. JIA patients had the following disease subtypes: 15 had persistent oligoarthritis, 13 had extended oligoarthritis, 4 had systemic onset, and 4 had polyarthritis. The mean age of the JIA patients was 10.4 years (range 1.7–39 years), and the mean disease duration was 5.3 years (range 0.2–18.6 years). Eleven patients (31%) were receiving methotrexate, 22 (61%) were receiving nonsteroidal antiinflammatory agents, and 2 (6%) were receiving oral prednisolone at the time of sampling. All patients attended the Great Ormond Street Hospital in London. The study had approval from the local ethics review committee. Full informed consent was obtained from the parents of each child.

All patients had active disease at the time of sampling, in that they required therapeutic joint aspiration and then intraarticular injection of steroid. Synovial tissue samples (n = 3) were collected at the time of synovectomy or arthroplasty and were snap frozen until further use. Paired samples of peripheral blood and synovial fluid were obtained at the time of clinically indicated arthrocentesis. Samples were processed within 1 hour of collection from the patient. Peripheral blood mononuclear cells (PBMCs) were isolated by standard Ficoll-Hypaque density centrifugation. For preparation of synovial fluid mononuclear cells (SFMCs), samples were first treated with 10 units/ml of hyaluronidase (Sigma, Poole, UK) for 30 minutes at 37°C, before density gradient isolation.

For analysis of cytokine production by T cells, the SFMCs or PBMCs were cultured for 3 hours in the presence of 50 ng/ml of phorbol myristate acetate (PMA), 500 ng/ml of ionomycin, and 5 μg/ml of Brefeldin A before analysis by intracellular cytokine staining and flow cytometry, as described elsewhere (3). For migration assays, purified CD4+ T cells were first prepared using magnetic beads (Miltenyi Biotec, Surrey, UK) according to the manufacturer's instructions. Purity of the CD4+ cells was always >92%.

### Analysis by flow cytometry

Standard 5-color flow cytometry was performed for surface markers using antibodies against the following human proteins with fluorescent labels: phycoerythrin (PE)–Cy7–labeled CD3 (SouthernBiotech, Birmingham, AL), PE-labeled CD25 (Dako, Ely, UK), fluorescein isothiocyanate (FITC)–labeled CD45RO (Serotec, Oxford, UK), PE-labeled CD8 (Dako), and FITC-labeled T cell receptor γ/δ (TCRγ/δ), peridinin chlorophyll A protein–labeled CD4, PE-labeled CCR4, and PE-labeled CCR6 (all from BD PharMingen, Oxford, UK).

For intracellular staining with antibodies against PE-labeled human IL-4, FITC-labeled IFNγ (both from BD PharMingen), Alexa Fluor 647–labeled IL-17A (eBioscience, San Diego, CA), and PE-labeled IL-22 (R&D Systems, Abingdon, UK), cells were first fixed in 4% paraformaldehyde (Sigma) in phosphate buffered saline and permeabilized in 0.1% saponin (Sigma); antibodies and wash buffer for intracellular staining also contained 0.1% saponin. Staining with allophycocyanin-labeled anti-FoxP3 was performed according to the manufacturer's instructions (eBioscience).

Flow cytometric data were collected with a Cyan ADP flow cytometer (Dako); 100,000 to 200,000 events were collected for each condition, and cells were gated by their light scatter properties. Data were analyzed using FlowJo software (Tree Star, Ashland, OR).

### Immunohistochemical staining of synovial tissue

Cryosections (7μ) were cut, fixed in acetone, and stained by standard immunohistochemistry methods using goat anti-human IL-22 (Santa Cruz Biotechnology, Santa Cruz, CA), goat anti-human IL-17 (R&D Systems), mouse anti-human CD4 (Novocastra, Newcastle upon Tyne, UK), isotype control antibody (Dako), or no primary antibody control, followed by biotinylated rabbit anti-goat Ig (Dako) or biotinylated donkey anti-mouse Ig (Chemicon, Billerica, MA) and a standard avidin–biotin complex protocol.

### Chemotaxis assay

Assays for chemotaxis to CCL20 (Insight Biotechnology, Wembley, UK) were performed using 5-μm–pore polycarbonate filters in a Transwell chamber (Corning Life Sciences, Schiphol-Rijk, The Netherlands) as described elsewhere (34), with a range of 10–1,000 ng/ml of CCL20 in RPMI 1640–0.5% bovine serum albumin (BSA). After 90 minutes, migrated cells were recovered, stimulated with PMA and ionomycin in the presence of Brefeldin A, and stained with anti-human IL-17, IFNγ, CD3, and CD4 as detailed above. Immediately prior to acquisition of data by flow cytometry, 20,000 fluorescence-activated cell sorter counting beads (Perfect Count; Cytognos, Salamanca, Spain) were added per sample. The number of cells acquired was standardized relative to the number of beads. To calculate the chemotactic index, the number of cells that migrated in response to chemokine was divided by the number of cells that migrated spontaneously to control medium (RPMI–0.5% BSA).

### Statistical analysis

Data were analyzed using SPSS v14.0 software (Chicago, IL) and GraphPad Prism software (San Diego, CA). Comparison of IL-17+ cell numbers between groups was analyzed by the Mann-Whitney U test. Linear regression was used to assess the relationship between IL-17+ cells and Treg cells. For this comparison, skewed data were log-transformed before analysis.

## RESULTS

### Enumeration of IL-17–producing T cells in the blood and joints of patients with JIA and correlations with clinical phenotype

**Figure 1:**
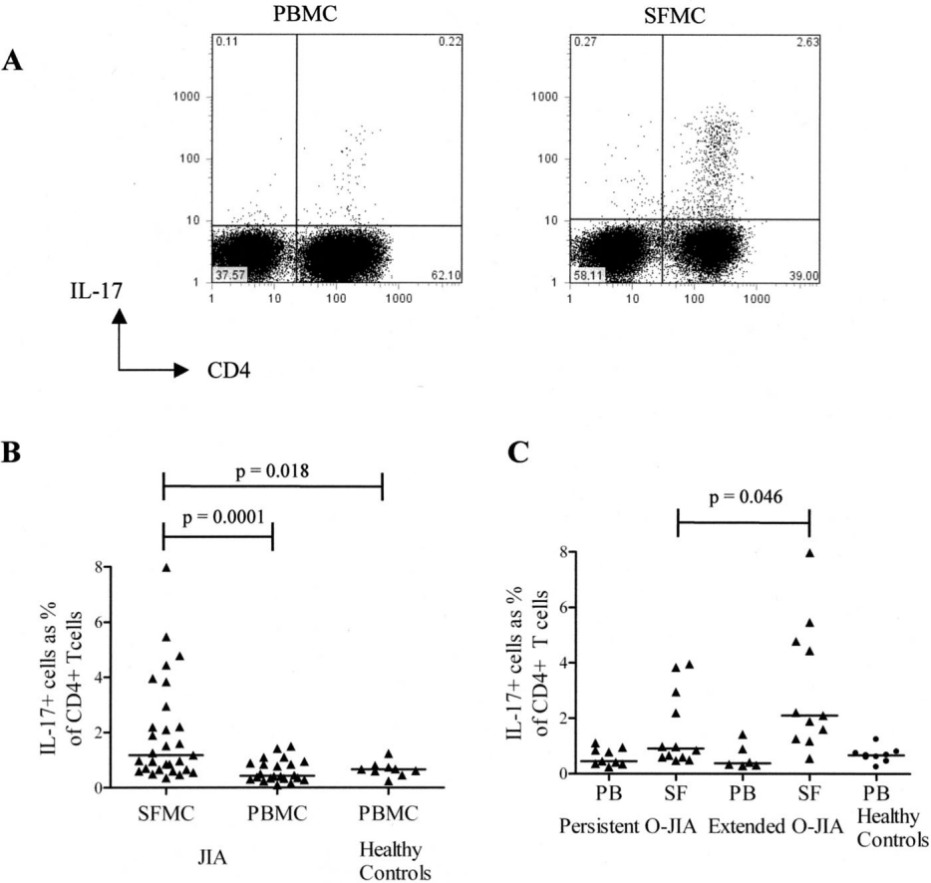
Enrichment of interleukin-17 (IL-17)–positive, CD4+ T cells in synovial fluid from patients with juvenile idiopathic arthritis (JIA) and correlation with clinical subtype. **A,** Representative dot plots of paired samples of peripheral blood mononuclear cells (PBMCs) and synovial fluid mononuclear cells (SFMCs) from a patient with JIA. Cells were stained for surface expression of CD3 and CD4 and then for intracellular expression of IL-17 and were analyzed by flow cytometry, gated on live lymphocytes and CD3+ cells. Numbers in each compartment are the percentage of cells. **B,** Numbers of IL-17+ cells as a percentage of CD4+ T cells in SFMCs from JIA patients (n = 28), PBMCs from JIA patients (n = 22), and PBMCs from healthy controls (n = 9). Bars show the median. **C,** Numbers of IL-17+ cells as a percentage of CD4+ T cells in PBMCs (PB) and SFMCs (SF) from patients with persistent (n = 9 PBMC samples; n = 12 SFMC samples) and extended (n = 6 PBMC samples; n = 13 SFMC samples) oligoarticular JIA (O-JIA) and in PBMCs from healthy controls (n = 9). Bars show the median.

We analyzed by flow cytometry mononuclear cells derived from peripheral blood and synovial fluid obtained from children with JIA for the presence of IL-17–producing T cells. We found a clear enrichment of IL-17–producing T cells within the synovial fluid as compared with the peripheral blood of the JIA patients (Figure 1). The majority of IL-17+ T cells were within the CD4+,CD3+ population (Figure 1A). In PBMCs from JIA patients, the median number of IL-17–producing cells constituted 0.43% of the CD4+ T cell population (interquartile range [IQR] 0.3–0.9). This did not differ significantly from the IL-17+ T cell numbers in PBMCs from healthy controls (median 0.67%; IQR 0.5–0.8) (Figure 1B). In contrast, the number of IL-17+,CD4+ T cells in SFMCs was significantly increased (median 1.2%; IQR 0.7–2.6) as compared with that in PBMCs from both the JIA patients (*P* = 0.0001) and the controls (*P* = 0.018) (Figure 1B). Although this difference was seen across all subtypes of JIA, the degree of enrichment varied between clinical phenotypes.

Analysis of the number of IL-17+,CD4+ T cells by subtype revealed a relationship to disease severity. There was a significant enrichment of IL-17+ T cells in the joints of children with extended oligoarticular JIA, the more severe form of JIA (median 2.1; IQR 1.3–4.8), as compared with persistent oligoarticular JIA, the milder form of the disease, which is self-remitting (median 0.91; IQR 0.6–2.8 [*P* = 0.046]) (Figure 1C).

**Figure 2:**
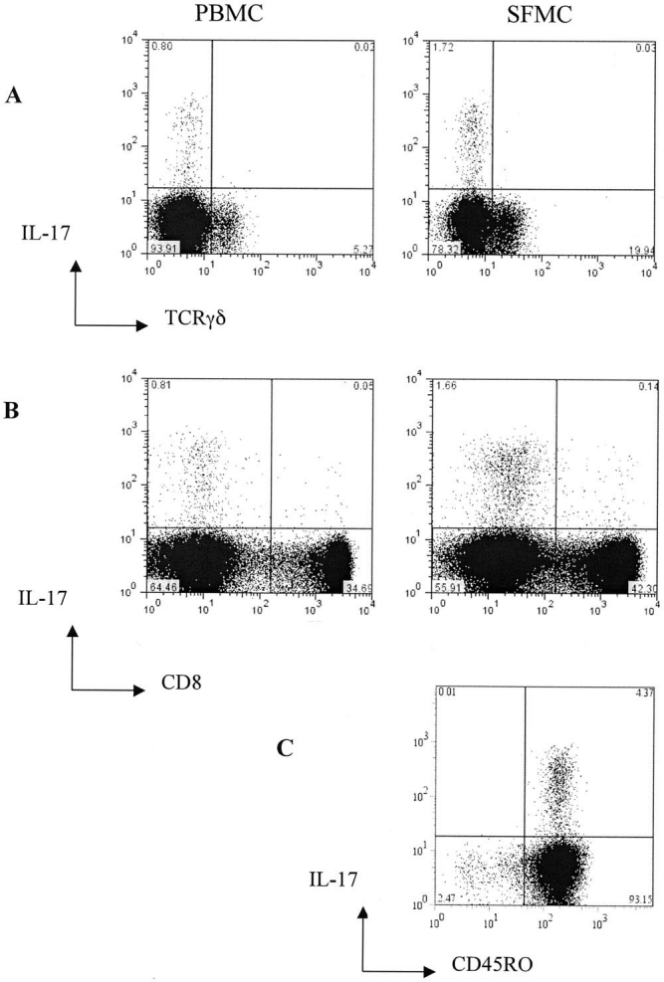
Characterization of interleukin-17 (IL-17)–positive T cells in patients with juvenile idiopathic arthritis (JIA). Peripheral blood mononuclear cells (PBMCs) and synovial fluid mononuclear cells (SFMCs) from JIA patients were analyzed by flow cytometry. **A** and **B,** Representative dot plots of paired PBMCs and SFMCs stained for CD3, IL-17, and T cell receptor γ/δ (TCRγ/δ) (**A**) as well as for CD3, IL-17, and CD8 (**B**), both gated on live lymphocytes and CD3+ cells. **C,** Representative dot plot of JIA SFMCs stained for CD3, CD4, CD45RO, and IL-17 gated on live lymphocytes, CD3+ cells, and CD4+ cells. Numbers in each compartment are the percentage of cells.

No IL-17–producing cells were demonstrated in the TCRγ/δ population, and few CD8+ T cells were found to be IL-17+ in either the JIA patients or the controls (Figures 2A and B). Consistent with the demonstration that IL-17+ T cells reside within the memory pool (27,35), IL-17+,CD4+ cells from both compartments (peripheral blood and joint) all expressed CD45RO (Figure 2C).

### Inverse relationship between IL-17+ and FoxP3+ T cells in patients with JIA

**Figure 3:**
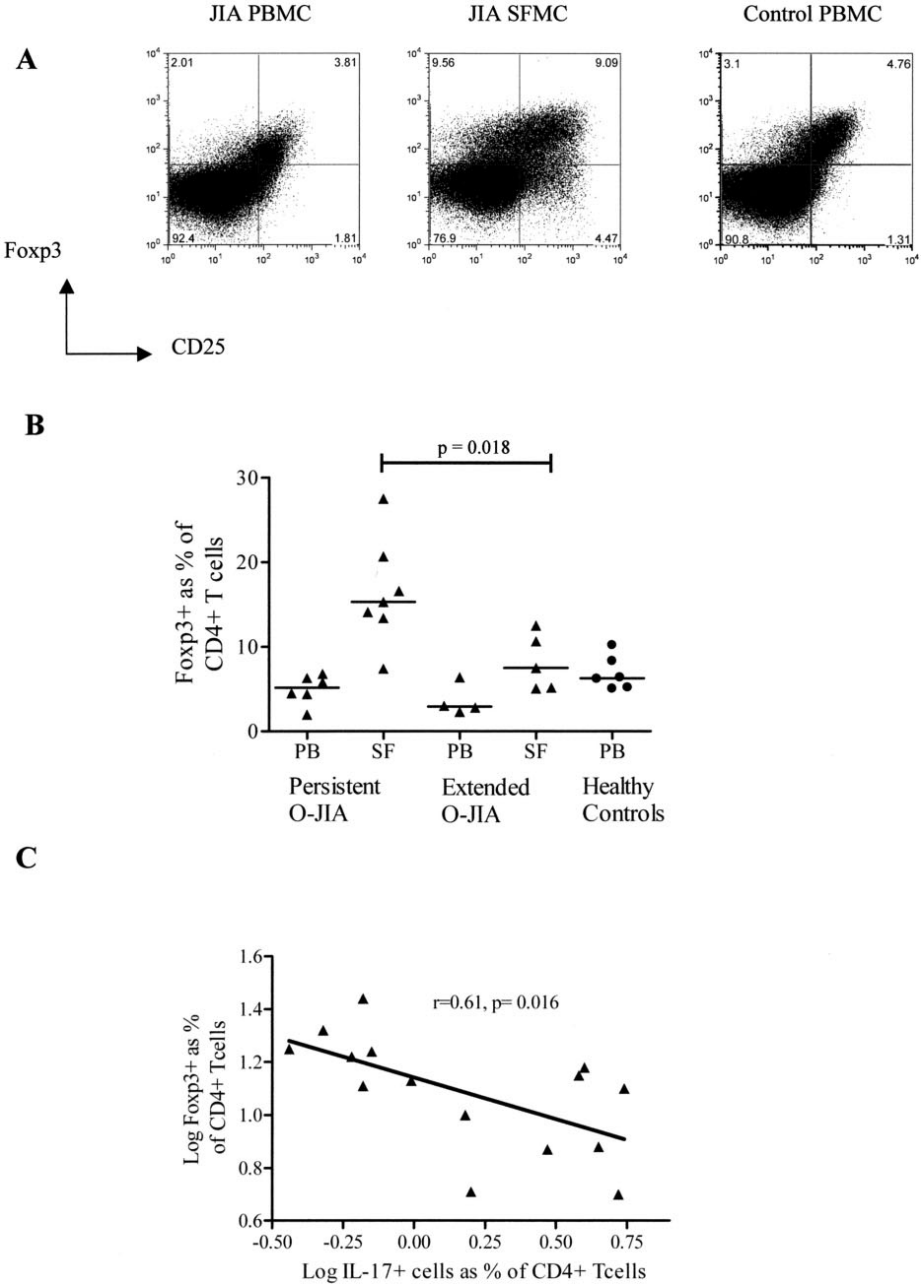
Inverse relationship between the number of synovial forkhead box P3 (FoxP3)–positive regulatory T cells and interleukin-17 (IL-17)–positive, CD4+ T cells. **A,** Representative dot plots of paired samples of peripheral blood mononuclear cells (PBMCs) and synovial fluid mononuclear cells (SFMCs) from a patient with juvenile idiopathic arthritis (JIA) and of samples of PBMCs from a healthy control subject. Cells were stained for surface expression of CD3, CD4, and CD25 and then for intracellular expression of FoxP3 and were analyzed by flow cytometry, gated on live lymphocytes and CD3+,CD4+ cells. Numbers in each compartment are the percentage of cells. **B,** Numbers of FoxP3+ cells as a percentage of CD4+ T cells in PBMCs and SFMCs from patients with persistent (n = 8) and extended (n = 5) oligoarticular JIA (O-JIA) and in PBMCs from 6 healthy controls. Bars show the median. **C,** Comparison of the numbers of IL-17+ and FoxP3+ cells as percentages of CD4+ T cells in SFMCs from 15 JIA patients. Linear regression was performed on log-transformed data.

Since it is known that the numbers of Treg cells in the joint also correlate with different clinical phenotypes of JIA (31,32), we next examined the relationship between Treg cells and Th17 cells in the context of this autoimmune disease. Previous studies using high levels of surface expression of CD25 to identify Treg cells showed that these cells expressed high levels of messenger RNA for the FoxP3 transcription factor and confirmed their suppressive function in vitro (31). In the present study, we used protein expression of FoxP3 as analyzed by flow cytometry to identify Treg cells. We confirmed that in the joint, where many T cells are highly activated, not all CD25^bright^ cells coexpressed FoxP3, while some FoxP3+ cells resided in the CD25^dim^ or CD25– population (Figure 3A).

Using FoxP3 protein expression to identify synovial Treg cells in samples from patients with different clinical subtypes of JIA, we confirmed our previous findings of a significant difference in the number of Treg cells in the joints of patients with persistent oligoarticular JIA and those with extended oligoarticular JIA (*P* = 0.018) (Figure 3B). Remarkably, the simultaneous analysis for IL-17+,CD4+ T cells and FoxP3+ Treg cells showed that these 2 populations exist in an inverse relationship at the site of inflammation (*P* = 0.016) (Figure 3C). No analogous relationship was observed in PBMCs from either the patients or the controls, and no cells expressed both FoxP3 and IL-17.

### Chemokine receptor analysis of IL-17+ T cells from patients with JIA

**Figure 4:**
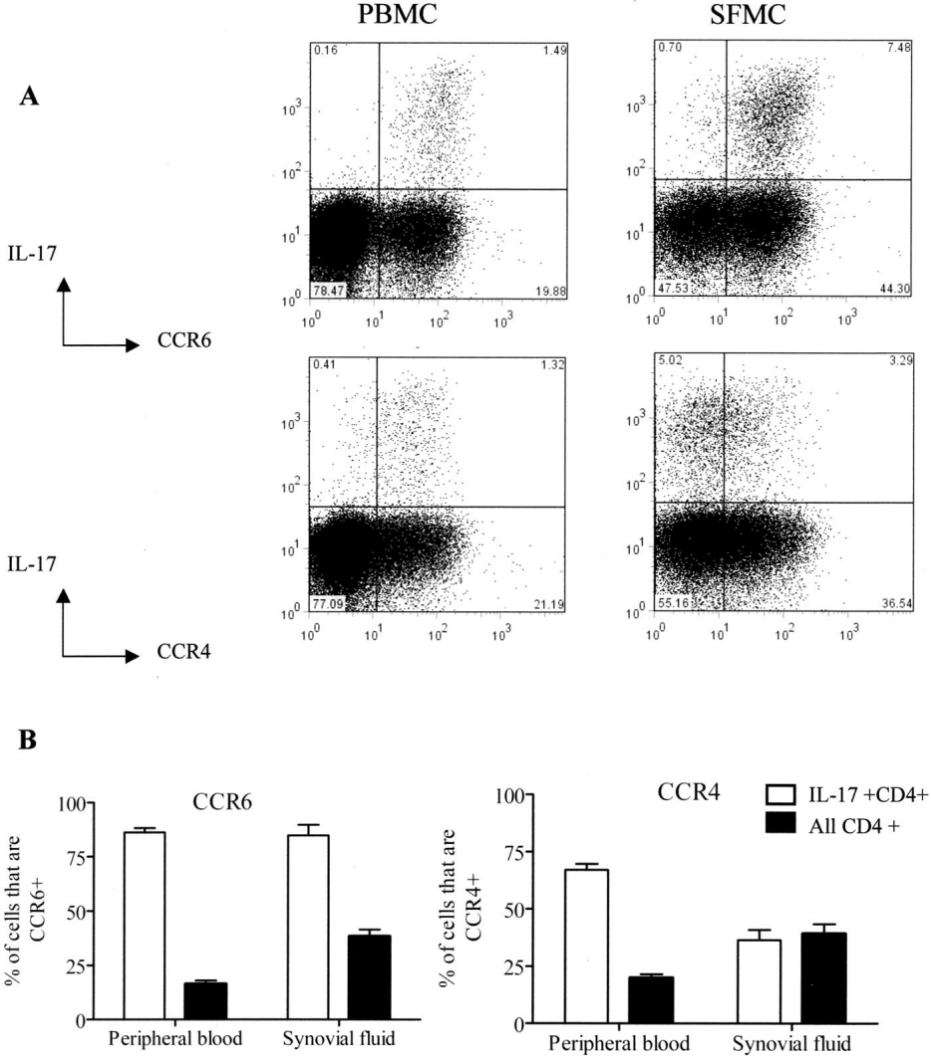
Divergent patterns of chemokine receptor CCR4 expression of interleukin-17 (IL-17)–positive, CD4+ T cells in peripheral blood mononuclear cells (PBMCs) and synovial fluid mononuclear cells (SFMCs) from patients with juvenile idiopathic arthritis (JIA). **A,** Representative dot plots of paired PBMCs and SFMCs from JIA patients, showing CCR6 and CCR4 expression on IL-17+,CD4+ T cells. Cells were analyzed by flow cytometry, gated on live lymphocytes and CD4+ cells. Numbers in each compartment are the percentage of cells. Results are representative of 1 experiment of 5 experiments performed. **B,** Percentage of all CD4+ T cells and IL-17+,CD4+ T cells expressing CCR6 and CCR4 in the peripheral blood and synovial fluid of 5 patients with JIA. Values are the mean and SEM.

We next considered the chemotactic influences that could affect the recruitment of IL-17+ T cells to sites of inflammation. We have previously shown high levels of expression of the chemokine receptors CCR5 and CXCR3 on synovial T cells in JIA (3). Recently reported data suggest that human Th17 cells express CCR6 and CCR4, but that a population of cells that produce both IFNγ and IL-17 preferentially expresses CCR6 and CXCR3 (27). We therefore investigated the expression of these receptors on Th17 cells in JIA. Analysis of the expression of CCR6 confirmed that a high proportion of IL-17–producing T cells from both the peripheral blood and joint expressed CCR6, as compared with the CD4+ T cell population as a whole (Figures 4A and B). In contrast, IL-17+ T cells from the joint expressed lower levels of CCR4 than did their counterparts (IL-17+,CD4+ T cells) from the blood (Figures 4A and B). Interestingly, FoxP3+ Treg cells in the JIA joint also expressed high levels of both CCR4 (31) and CCR6 (data not shown).

### Coexpression of inflammatory cytokines by IL-17+,CD4+ T cells from synovial fluid and synovial tissue obtained from patients with JIA

Given the apparent discrepancy in the expression of CCR4 on IL-17+ synovial T cells compared with that on IL-17+ peripheral blood T cells and the recent demonstration that cells with a CCR6+,CCR4– phenotype may include Th1 cells that also produce IL-17, we hypothesized that synovial IL-17+ T cells in JIA would include a population of cells that secrete both IL-17 and IFNγ. We therefore investigated the functional diversity of these cells.

**Figure 5:**
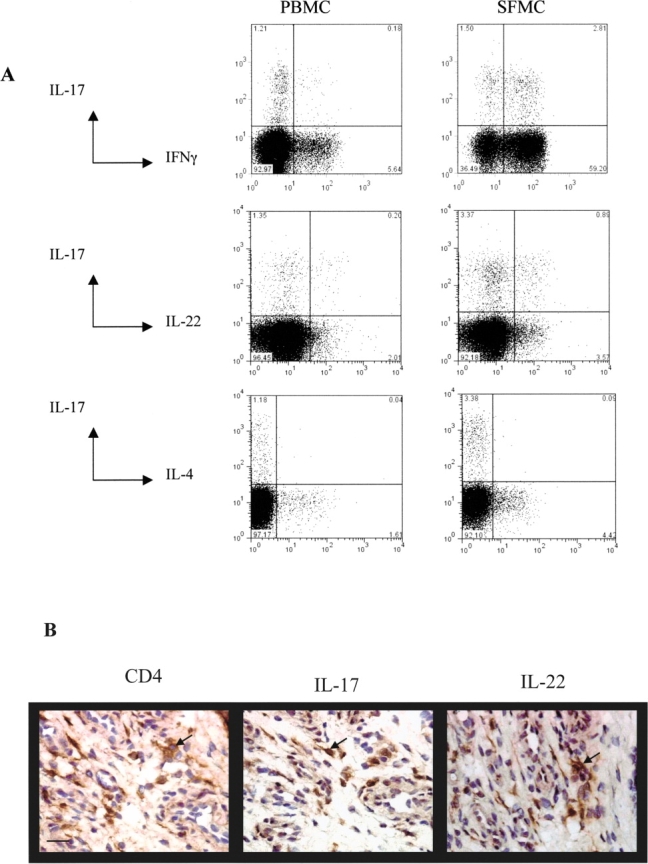
Dysregulated cytokine expression by interleukin-17 (IL-17)–positive, CD4+ T cells in the synovial fluid of juvenile idiopathic arthritis (JIA) patients. **A,** Representative dot plots of paired samples of peripheral blood mononuclear cells (PBMCs) and synovial fluid mononuclear cells (SFMCs) from patients with JIA. Cells were stained for intracellular cytokines IL-17, interferon-γ (IFNγ), IL-22, and IL-4 and analyzed by flow cytometry, gated on live lymphocytes and CD3+,CD4+ cells. Numbers in each compartment are the percentage of cells. **B,** Immunohistologic localization of IL-17 and IL-22 in JIA synovial tissue. Frozen sections of synovial biopsy tissue were stained for CD4, IL-17, and IL-22 by standard immunohistochemical techniques. Tissue sections show typical hypertrophied synovium and dense inflammatory infiltrate. Positive cells expressing the marker of interest are stained brown; **arrow** shows typical positive cells. Bar = 20 μm.

IL-22 has been demonstrated to be produced by Th17 cells (36,37) and has been implicated in RA (38). Analysis of IL-22 and IFNγ production in IL-17+, CD4+ T cells showed that a significant proportion of cells that produced IL-17 also produced IL-22 and similarly demonstrated coexpression of IL-17 and IFNγ (Figure 5A). The number of these “double cytokine (IL-17 and IFNγ)–positive” T cells were consistently higher in SFMC T cells than in PBMC T cells from either the patients or the controls. Thus, IL-17 and IFNγ double-positive CD4+ T cells constituted a median of 46.8% (IQR 26.3–59.1) of IL-17–producing cells in the joint (n = 14), as compared with a median of 12.0% (IQR 4.4–15.5) in control PBMCs (n = 9) and 16.1% (IQR 8.7–23.9) in JIA PBMCs (n = 14), both of which were significantly different from the median in SFMCs (*P* < 0.001 for each comparison). In contrast, production of the Th2 cytokine IL-4 was mutually exclusive with IL-17, with expression of these cytokines in discrete and separate populations in both the blood and the joint (n = 5) (Figure 5A).

To demonstrate the expression of both IL-17 and IL-22 at the site of disease, synovial tissue was analyzed by immunohistochemistry. This analysis confirmed that both IL-17 and IL-22 were expressed on infiltrating CD4+ cells within the inflamed synovial tissue (Figure 5B).

### Functional migration of Th17 cells to CCL20

**Figure 6:**
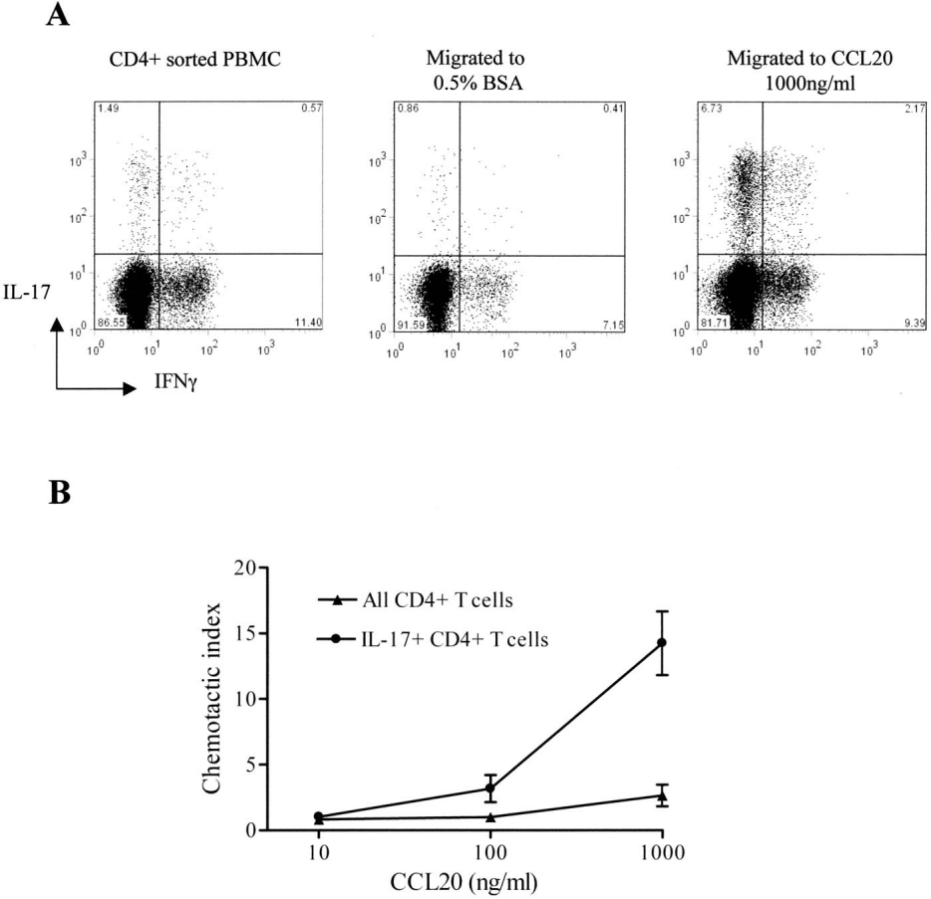
Preferential migration of interleukin-17 (IL-17)–positive, CD4+ T cells to the CCR6 ligand CCL20 as compared with CD4+ T cells. Purified CD4+ T cells from patients with juvenile idiopathic arthritis (JIA) and healthy controls were migrated to 10 ng/ml, 100 ng/ml, or 1,000 ng/ml of CCL20 or RPMI 1640–0.5% bovine serum albumin (BSA). **A,** Cells from healthy controls analyzed by intracellular cytokine staining for IL-17 and interferon-γ (IFNγ) before (left) and after migration to control medium (RPMI 1640–0.5% BSA) (middle) or 1,000 ng/ml of CCL20 (right). Cells were analyzed by flow cytometry, gated on live CD3+,CD4+ cells. Numbers in each compartment are the percentage of cells. Results are representative of 1 experiment of 5 experiments performed. **B,** Response of IL-17+,CD4+ T cells and all CD4+ T cells to a titration of the CCL20 gradient. Data are expressed as the chemotactic index (ratio of cells migrating to CCL20 divided by cells migrating to control medium). Values are the mean ± SEM of 5 experiments.

The ability of CD4+ T cells from PBMCs to migrate in response to the CCR6 ligand CCL20 was tested using standard migration assays. Migrated cells were then assayed for the production of IL-17 and IFNγ as described above (Figure 6). IL-17+ T cells from both the patients and the controls were enriched in cells that migrated toward CCL20 (Figure 6A) and, as expected, the response titrated across a range of concentrations (Figure 6B). Under standard conditions using 100 ng/ml of CCL20 ligand, the mean chemotactic index for IL-17+,CD4+ T cells from PBMCs did not differ significantly between healthy controls (mean ± SD 3.2 ± 2.3) and JIA patients (3.8 ± 0.9).

## DISCUSSION

In this report, we present the first detailed analysis of IL-17–producing T cells in JIA and their relationship to FoxP3+ regulatory T cells. Multiple proinflammatory mediators contribute to the tissue damage seen in inflammatory arthritis in humans, both in adults and in children. These include TNFα, IL-1, IL-6, RANKL, and chemokines, many of which have been shown to be present at high levels in synovial fluid of both RA and JIA patients (39,40). It is now well established that inflammatory reactions may “coexist” with a regulatory response. We propose that, at least in JIA, the balance between proinflammatory factors and immune regulation has a profound impact on clinical phenotype. Thus, in patients with the mild, remitting phenotype of JIA known as persistent oligoarticular JIA, we and other investigators have previously seen that Treg cells are present at higher numbers in the inflamed joint than in patients with more severe disease (31,32). We have now extended these findings by our analysis of IL-17–producing T cells in JIA.

Despite the success of treatment of childhood arthritis with TNFα blockade, a proportion of children with JIA fail to achieve remission with standard therapies. There is renewed interest in novel targets for therapeutic blockade in arthritis, including IL-17 family members, which both synergize with, and have effects independent of, TNFα and IL-1 (41), as well as IL-17 receptors (42,43). IL-17 has been demonstrated in the joints of patients with RA and patients with JIA, but to date, few studies have characterized the cells that produce IL-17 in humans with arthritis. In animal model systems, including collagen-induced arthritis, IL-17 has been shown to be critical and sufficient to produce disease in the absence of IFNγ (44), and several reports suggest that IFNγ can even be protective, perhaps by inhibiting the development of Th17 cells (35,45). Initial reports suggest that T cells that secrete IL-17 can be detected in the peripheral blood of healthy donors and may reside in more than 1 subpopulation (27), which may be distinguished by their differential expression of specific chemokine receptors. It is now vital to have a detailed analysis of IL-17–producing T cells in human disease in order to better understand their role in disease pathogenesis and to design appropriately targeted therapies.

In this study of 36 children with JIA, we showed that T cells that produce IL-17 are highly enriched within the inflamed joints of our patients, and we demonstrated these cells at the site of disease within the joint. IL-17 was produced predominantly by CD4+ T cells in the joint, and these cells were present at significantly higher numbers in the joint than in either the paired blood samples from JIA patients or the blood of healthy controls. The IL-17+ T cells were uniformly within the memory CD4+,CD45RO+ population. Previously, IL-17 was estimated by staining studies to be produced by ∼1% of synovial tissue T cells. Our flow cytometric data from synovial fluid T cell analyses are consistent with this estimate.

We also demonstrated that these IL-17+ cells were present in higher numbers in the joints of patients with extended oligoarticular JIA, the more severe phenotype, than in those with persistent oligoarticular JIA, the mild form of disease. These results suggest that IL-17+ T cells relate directly to clinical phenotype in JIA. Given previous findings on the role of Treg cells in JIA and evidence that Treg cells and Th17 cells may share developmental pathways, we also analyzed Treg cell numbers in these patients and found a reciprocal relationship between Treg cell and IL-17+ T cell numbers in the joint. This suggests that within the microenvironment of the joint, an integrated set of factors that lead to the recruitment or survival of these 2 T cell populations will lead to a predominance of either Treg cells or IL-17+ T cells.

Several studies have demonstrated that the development of murine Th17 cells and Treg cells may be interrelated, both being dependent on TGFβ, yet reciprocally influenced by cytokines, such as IL-6, IL-1 (which switch toward Th17) (8), as well as IL-2 and the vitamin A metabolite all-*trans*-retinoic acid, both of which switch toward Treg cells (46,47). However, the majority of published studies were performed using highly purified naive T cells, and the data are not readily extrapolated to apply to sites of chronic inflammation in human disease. Findings of a recent study suggest that Treg cells themselves may convert to Th17 cells in the presence of IL-6 (48). It is known that Treg cells can themselves limit expansion of Th17 cells, both in vitro (12) and in vivo (45). Analyses of IL-1, IL-6, and IL-2 at sites of tissue inflammation will clearly be of importance. Our preliminary data (Nistala K, Moncrieffe H: unpublished observations) suggest that there is a correlation between the levels of IL-6 and the levels of IL-17 within the joint. Emerging data in human studies suggest that the “rules” governing human Th17 cells may diverge from those that govern mouse data; for example, in humans, Th17 cell generation appears to be driven by IL-6 or IL-1β, but not TGFβ, and IL-2 fails to inhibit Th17 cell generation (35). The reciprocal effects of all-*trans*-retinoic acid on the development of Th17 and Treg cells have yet to be tested in humans.

It has previously been shown that RA synovial fluid T cells may coexpress IFNγ and IL-17 (49). T cell clones grown from the joint may produce both IFNγ and IL-17 (50), although these cells were not fully characterized ex vivo. In patients with Lyme arthritis, IL-17+ T cells have been demonstrated within both the IFNγ+ and TNFα+ populations of CD4+ T cells (29). Evidence showing that human Th17 cells typically express CCR4 and CCR6 and that these are enriched in memory cells specific for fungal antigens, such as *Candida albicans*, whereas a fraction of Th1 cells that produce both IFNγ and IL-17 typically express CCR6 and CXCR3, has been reported (27). In our study, we confirmed that in the synovial fluid of JIA patients and in the blood of both JIA patients and controls, the majority of IL-17+ T cells express CCR6. Migration of CD4+ T cells toward a CCL20 gradient demonstrated an enrichment of IL-17+ T cells in both patients and controls.

Interestingly, the CCR6 ligand CCL20 (previously called macrophage inflammatory protein 3α) is itself produced by synovial explants in response to IL-17 (51). In addition, CCL20 has been shown to be up-regulated in human Th17 cells and, in fact, to be a defining feature of the Th17 “signature,” along with IL-17 and IL-22 (14). The high levels of this chemokine in the synovial fluid of patients with arthritis may contribute to the enrichment of IL-17–producing cells within the joint (52). Our data raise the possibility that Th17 cell migration to the joint may set up a positive feedback loop for further recruitment through the autologous production of CCL20.

In PBMCs, the expression of CCR4 on IL-17+ T cells mirrored that of CCR6. However, within the joint, IL-17+ T cells had a variable expression of CCR4. These data suggested that a CCR6+,CCR4– population was enriched within the inflamed joint and led us to further characterize other cytokines produced by these cells. IL-17+ T cells in peripheral blood were clearly distinct from either Th1 cells (defined by IFNγ production) or Th2 cells (defined by IL-4 production). However, again, the site of inflammation showed a dysregulation, in that typically, more than half of those T cells producing IL-17 were within the IFNγ-producing population. The small IL-4+ population of T cells within the joint was distinct from the IL-17+ cells. We have previously shown that Th1,IFNγ+,CXCR3+,CD4+ T cells are highly enriched in the synovium in JIA (3). The findings of our current study suggest that within this proinflammatory population, a proportion of these cells also make the highly active cytokine IL-17.

This study is the first to demonstrate that IL-17–expressing T cells are highly enriched in the inflamed joints of children with arthritis and that their numbers directly correlate with the clinical phenotype. These IL-17+ T cells include populations that also produce IL-22 and IFNγ, but not IL-4. The synovial IL-17+ T cells all express CCR6, but have a variable expression of CCR4. We propose that despite the highly enriched numbers of IL-17+ T cells in the joint compared with the blood, immune regulation also has a direct impact at sites of inflammation in childhood arthritis, since we found a reciprocal relationship between IL-17+ T cells and FoxP3+ Treg cells. This suggests an explanation for the persistence of arthritic damage to the joint despite the presence of regulation. The elucidation of factors that control this balance and reciprocal relationship is crucial to our understanding of ongoing autoimmune pathology in arthritis and, therefore, to our ability to intervene therapeutically in favor of regulation.

## AUTHOR CONTRIBUTIONS

Dr. Wedderburn had full access to all of the data in the study and takes responsibility for the integrity of the data and the accuracy of the data analysis.

**Study design.** Nistala, Moncrieffe, Hunter, Wedderburn.

**Acquisition of data.** Nistala, Moncrieffe, Newton, Varsani, Wedderburn.

**Analysis and interpretation of data.** Nistala, Moncrieffe, Hunter, Wedderburn.

**Manuscript preparation.** Nistala, Wedderburn.

**Statistical analysis.** Nistala, Wedderburn.

## References

[1] Mosmann TR, Cherwinski H, Bond MW, Giedlin MA, Coffman RL (1986). Two types of murine helper T cell clone. I. Definition according to profiles of lymphokine activities and secreted proteins. J Immunol.

[2] Dolhain RJ, van der Heiden AN, ter Haar NT, Breedveld FC, Miltenburg AM (1996). Shift toward T lymphocytes with a T helper 1 cytokine-secretion profile in the joints of patients with rheumatoid arthritis. Arthritis Rheum.

[3] Wedderburn LR, Robinson N, Patel A, Varsani H, Woo P (2000). Selective recruitment of polarized T cells expressing CCR5 and CXCR3 to the inflamed joints of children with juvenile idiopathic arthritis. Arthritis Rheum.

[4] Mangan PR, Harrington LE, O'Quinn DB, Helms WS, Bullard DC, Elson CO (2006). Transforming growth factor-β induces development of the T_H_17 lineage. Nature.

[5] Harrington LE, Mangan PR, Weaver CT (2006). Expanding the effector CD4 T-cell repertoire: the Th17 lineage. Curr Opin Immunol.

[6] Weaver CT, Harrington LE, Mangan PR, Gavrieli M, Murphy KM (2006). Th17: an effector CD4 T cell lineage with regulatory T cell ties. Immunity.

[7] Park H, Li Z, Yang XO, Chang SH, Nurieva R, Wang YH (2005). A distinct lineage of CD4 T cells regulates tissue inflammation by producing interleukin 17. Nat Immunol.

[8] Veldhoen M, Hocking RJ, Atkins CJ, Locksley RM, Stockinger B (2006). TGFβ in the context of an inflammatory cytokine milieu supports de novo differentiation of IL-17-producing T cells. Immunity.

[9] Ivanov II, McKenzie BS, Zhou L, Tadokoro CE, Lepelley A, Lafaille JJ (2006). The orphan nuclear receptor RORγt directs the differentiation program of proinflammatory IL-17+ T helper cells. Cell.

[10] Nakae S, Nambu A, Sudo K, Iwakura Y (2003). Suppression of immune induction of collagen-induced arthritis in IL-17-deficient mice. J Immunol.

[11] Langrish CL, Chen Y, Blumenschein WM, Mattson J, Basham B, Sedgwick JD (2005). IL-23 drives a pathogenic T cell population that induces autoimmune inflammation. J Exp Med.

[12] Bettelli E, Carrier Y, Gao W, Korn T, Strom TB, Oukka M (2006). Reciprocal developmental pathways for the generation of pathogenic effector T_H_17 and regulatory T cells. Nature.

[13] Acosta-Rodriguez EV, Napolitani G, Lanzavecchia A, Sallusto F (2007). Interleukins 1β and 6 but not transforming growth factor-β are essential for the differentiation of interleukin 17-producing human T helper cells. Nat Immunol.

[14] Wilson NJ, Boniface K, Chan JR, McKenzie BS, Blumenschein WM, Mattson JD (2007). Development, cytokine profile and function of human interleukin 17-producing helper T cells. Nat Immunol.

[15] Kotake S, Udagawa N, Takahashi N, Matsuzaki K, Itoh K, Ishiyama S (1999). IL-17 in synovial fluids from patients with rheumatoid arthritis is a potent stimulator of osteoclastogenesis. J Clin Invest.

[16] Chabaud M, Durand JM, Buchs N, Fossiez F, Page G, Frappart L (1999). Human interleukin-17: a T cell–derived proinflammatory cytokine produced by the rheumatoid synovium. Arthritis Rheum.

[17] De Jager W, Hoppenreijs EP, Wulffraat NM, Wedderburn LR, Kuis W, Prakken BJ (2007). Blood and synovial fluid cytokine signatures in patients with juvenile idiopathic arthritis: a cross-sectional study. Ann Rheum Dis.

[18] Jovanovic DV, Di Battista JA, Martel-Pelletier J, Jolicoeur FC, He Y, Zhang M (1998). IL-17 stimulates the production and expression of proinflammatory cytokines, IL-β and TNF-α, by human macrophages. J Immunol.

[19] Chabaud M, Fossiez F, Taupin JL, Miossec P (1998). Enhancing effect of IL-17 on IL-1-induced IL-6 and leukemia inhibitory factor production by rheumatoid arthritis synoviocytes and its regulation by Th2 cytokines. J Immunol.

[20] Katz Y, Nadiv O, Beer Y (2001). Interleukin-17 enhances tumor necrosis factor a–induced synthesis of interleukins 1,6, and 8 in skin and synovial fibroblasts: a possible role as a “fine-tuning cytokine” in inflammation processes. Arthritis Rheum.

[21] Varsani H, Patel A, van Kooyk Y, Woo P, Wedderburn LR (2003). Synovial dendritic cells in juvenile idiopathic arthritis (JIA) express receptor activator of NF-κB (RANK). Rheumatology (Oxford).

[22] Masi L, Simonini G, Piscitelli E, Del Monte F, Giani T, Cimaz R (2004). Osteoprotegerin (OPG)/RANK-L system in juvenile idiopathic arthritis: is there a potential modulating role for OPG/RANK-L in bone injury?. J Rheumatol.

[23] Gravallese EM, Manning C, Tsay A, Naito A, Pan C, Amento E (2000). Synovial tissue in rheumatoid arthritis is a source of osteoclast differentiation factor. Arthritis Rheum.

[24] Kotake S, Udagawa N, Hakoda M, Mogi M, Yano K, Tsuda E (2001). Activated human T cells directly induce osteoclastogenesis from human monocytes: possible role of T cells in bone destruction in rheumatoid arthritis patients. Arthritis Rheum.

[25] Chabaud M, Garnero P, Dayer JM, Guerne PA, Fossiez F, Miossec P (2000). Contribution of interleukin 17 to synovium matrix destruction in rheumatoid arthritis. Cytokine.

[26] Chabaud M, Lubberts E, Joosten L, van den Berg W, Miossec P (2001). IL-17 derived from juxta-articular bone and synovium contributes to joint degradation in rheumatoid arthritis. Arthritis Res.

[27] Acosta-Rodriguez EV, Rivino L, Geginat J, Jarrossay D, Gattorno M, Lanzavecchia A (2007). Surface phenotype and antigenic specificity of human interleukin 17-producing T helper memory cells. Nat Immunol.

[28] Aarvak T, Chabaud M, Kallberg E, Miossec P, Natvig JB (1999). Change in the Th1/Th2 phenotype of memory T-cell clones from rheumatoid arthritis synovium. Scand J Immunol.

[29] Infante-Duarte C, Horton HF, Byrne MC, Kamradt T (2000). Microbial lipopeptides induce the production of IL-17 in Th cells. J Immunol.

[30] Petty RE, Southwood TR, Manners P, Baum J, Glass DN, Goldenberg J (2004). International League of Associations for Rheumatology classification of juvenile idiopathic arthritis: second revision, Edmonton, 2001. J Rheumatol.

[31] De Kleer IM, Wedderburn LR, Taams LS, Patel A, Varsani H, Klein M (2004). CD4^+^CD25^bright^ regulatory T cells actively regulate inflammation in the joints of patients with the remitting form of juvenile idiopathic arthritis. J Immunol.

[32] Ruprecht CR, Gattorno M, Ferlito F, Gregorio A, Martini A, Lanzavecchia A (2005). Coexpression of CD25 and CD27 identifies FoxP3+ regulatory T cells in inflamed synovia. J Exp Med.

[33] Pharoah DS, Varsani H, Tatham RW, Newton KR, de Jager W, Prakken BJ (2006). Expression of the inflammatory chemokines CCL5, CCL3 and CXCL10 in juvenile idiopathic arthritis, and demonstration of CCL5 production by an atypical subset of CD8+ T cells. Arthritis Res Ther.

[34] Iellem A, Mariani M, Lang R, Recalde H, Panina-Bordignon P, Sinigaglia F (2001). Unique chemotactic response profile and specific expression of chemokine receptors CCR4 and CCR8 by CD4^+^CD25^+^ regulatory T cells. J Exp Med.

[35] Amadi-Obi A, Yu CR, Liu X, Mahdi RM, Clarke GL, Nussenblatt RB (2007). T_H_17 cells contribute to uveitis and scleritis and are expanded by IL-2 and inhibited by IL-27/STAT1. Nat Med.

[36] Liang SC, Tan XY, Luxenberg DP, Karim R, Dunussi-Joannopoulos K, Collins M (2006). Interleukin (IL)-22 and IL-17 are coexpressed by Th17 cells and cooperatively enhance expression of antimicrobial peptides. J Exp Med.

[37] Zheng Y, Danilenko DM, Valdez P, Kasman I, Eastham-Anderson J, Wu J (2007). Interleukin-22, a T_H_17 cytokine, mediates IL-23-induced dermal inflammation and acanthosis. Nature.

[38] Ikeuchi H, Kuroiwa T, Hiramatsu N, Kaneko Y, Hiromura K, Ueki K (2005). Expression of interleukin-22 in rheumatoid arthritis: potential role as a proinflammatory cytokine. Arthritis Rheum.

[39] Feldmann M, Brennan FM, Maini RN (1996). The role of cytokines in rheumatoid arthritis. Annu Rev Immunol.

[40] Rooney M, Varsani H, Martin K, Lombard PR, Dayer JM, Woo P (2000). Tumour necrosis factor α and its soluble receptors in juvenile chronic arthritis. Rheumatology (Oxford).

[41] Koenders MI, Lubberts E, van de Loo FA, Oppers-Walgreen B, van den Bersselaar L, Helsen MM (2006). Interleukin-17 acts independently of TNF-α under arthritic conditions. J Immunol.

[42] Asquith DL, McInnes IB (2007). Emerging cytokine targets in rheumatoid arthritis. Curr Opin Rheumatol.

[43] Miossec P (2007). Interleukin-17 in fashion, at last: ten years after its description, its cellular source has been identified. Arthritis Rheum.

[44] Murphy CA, Langrish CL, Chen Y, Blumenschein W, McClanahan T, Kastelein RA (2003). Divergent pro-and antiinflammatory roles for IL-23 and IL-12 in joint autoimmune inflammation. J Exp Med.

[45] Lohr J, Knoechel B, Wang JJ, Villarino AV, Abbas AK (2006). Role of IL-17 and regulatory T lymphocytes in a systemic autoimmune disease. J Exp Med.

[46] Kryczek I, Wei S, Zou L, Altuwaijri S, Szeliga W, Kolls J (2007). Cutting edge: Th17 and regulatory T cell dynamics and the regulation by IL-2 in the tumor microenvironment. J Immunol.

[47] Mucida D, Park Y, Kim G, Turovskaya O, Scott I, Kronenberg M (2007). Reciprocal T_H_17 and regulatory T cell differentiation mediated by retinoic acid. Science.

[48] Xu L, Kitani A, Fuss I, Strober W (2007). Cutting edge: regulatory T cells induce CD4^+^CD25^–^Foxp3^–^ T cells or are self-induced to become Th17 cells in the absence of exogenous TGF-β. J Immunol.

[49] Page G, Sattler A, Kersten S, Thiel A, Radbruch A, Miossec P (2004). Plasma cell-like morphology of Th1-cytokine-producing cells associated with the loss of CD3 expression. Am J Pathol.

[50] Aarvak T, Chabaud M, Miossec P, Natvig JB (1999). IL-17 is produced by some proinflammatory Th1/Th0 cells but not by Th2 cells. J Immunol.

[51] Chabaud M, Page G, Miossec P (2001). Enhancing effect of IL-1, IL-17, and TNF-α on macrophage inflammatory protein-3α production in rheumatoid arthritis: regulation by soluble receptors and Th2 cytokines. J Immunol.

[52] Matsui T, Akahoshi T, Namai R, Hashimoto A, Kurihara Y (2001). Selective recruitment of CCR6-expressing cells by increased production of MIP-3α in rheumatoid arthritis. Clin Exp Immunol.

